# An open-label pilot trial assessing tolerability and feasibility of LSD microdosing in patients with major depressive disorder (LSDDEP1)

**DOI:** 10.1186/s40814-023-01399-8

**Published:** 2023-10-05

**Authors:** Carina Joy Donegan, Dimitri Daldegan-Bueno, Rachael Sumner, David Menkes, William Evans, Nicholas Hoeh, Frederick Sundram, Lisa Reynolds, Rhys Ponton, Alana Cavadino, Todd Smith, Partha Roop, Nathan Allen, Binu Abeysinghe, Darren Svirskis, Anna Forsyth, Mahima Bansal, Suresh Muthukumaraswamy

**Affiliations:** 1 Department of Psychological Medicine, Faculty of Medical and Health Sciences, University of Auckland, 2 Park Road, Grafton, Auckland, 1023 New Zealand; 2 School of Pharmacy, Faculty of Medical and Health Sciences, University of Auckland, 85 Park Road, Grafton, Auckland, 1023 New Zealand; 3 Department of Psychological Medicine, Faculty of Medical and Health Sciences, Waikato, Clinical Campus, University of Auckland, Pembroke Street, Hamilton, 3240 New Zealand; 4 Mana Health, 7 Ruskin St, Parnell, Auckland, 1052 New Zealand; 5 School of Population Health, Faculty of Medical and Health Sciences, University of Auckland, 2 Park Road, Grafton, Auckland, 1023 New Zealand; 6 Te Whatu Ora, Auckland, 1023 New Zealand; 7 Faculty of Engineering, University of Auckland, Auckland, 1023 New Zealand

**Keywords:** Microdosing, Lysergic acid diethylamide, Psychedelics, Major depressive disorder

## Abstract

**Background:**

Globally, an estimated 260 million people suffer from depression [1], and there is a clear need for the development of new, alternative antidepressant therapies. In light of problems with the tolerability and efficacy of available treatments [2], a global trend is emerging for patients to self-treat depression with microdoses of psychedelic drugs such as lysergic acid diethylamide (LSD) and psilocybin [3]. Beyond anecdotal reports from those who self-medicate in this way, few clinical trials have evaluated this practice. In our recently published phase 1 study in healthy volunteers [4], we determined that LSD microdosing was relatively safe and well tolerated in that cohort. Furthermore, the data demonstrated that conducting such microdosing trials is broadly feasible, with excellent adherence and compliance to the regimen observed. In this open-label pilot trial of patients with major depressive disorder (LSDDEP1), we will test the tolerability and feasibility of an 8-week regimen of LSD microdosing in this patient group prior to a larger subsequent randomised controlled trial (LSDDEP2).

**Methods:**

Twenty patients meeting the DSM-5 criteria for major depressive disorder will receive an 8-week LSD microdosing treatment regimen. The treatment protocol will use a sublingual formulation of LSD (MB-22001) delivered twice per week under a titration schedule using a dose of 5–15 µg. Tolerability will be assessed by quantifying the percentage of participants who withdraw from the trial due to adverse events attributable to the treatment regimen, while feasibility will be assessed by quantifying the percentage of attended clinic visits once enrolled. To determine whether there is any antidepressant response to the LSD microdosing regimen, MADRS scores will be assessed at baseline and 2, 4, 6, and 8 weeks after the commencement of the regimen.

**Discussion:**

The results of LSDDEP1 will provide valuable information regarding the tolerability and feasibility of a proposed LSD microdosing regimen in patients with MDD. Such information is critically important to optimise trial design prior to commencing a subsequent and more resource-intensive randomised controlled trial.

**Trial registration:**

ANZCTR, ACTRN12623000486628. Registered on 12 May 2023.

**Supplementary Information:**

The online version contains supplementary material available at 10.1186/s40814-023-01399-8.

## Background and rationale

Major depressive disorder (MDD) is the leading cause of global disability, with over 260 million people affected [1]. In Aotearoa/New Zealand, the jurisdiction of this study, approximately 6% of persons experience a depressive episode each year [2]. Depressive disorders cause significant detriment for the individual, their family (whānau), and society as a whole with significant social and economic impacts [2–4]. Despite this prevalence, current medical therapies are limited by slow onset and variable tolerability, with partial or total lack of efficacy in approximately one-third of patients [5]. Surveys have shown that while most New Zealanders who take antidepressants feel they are helpful for mood, many report problems with drug withdrawal (74%), sexual dysfunction (72%), weight gain (65%), and emotional numbing (65%); all of which negatively impact quality of life [6]. In Aotearoa/New Zealand, patients with depression form the “missing middle” identified by He Ara Oranga [7]. These patients are currently not being served adequately by mental health services and are, arguably, the population most in need of new treatment approaches. With the compounding effects of high depressive disorder prevalence and low efficacy of antidepressant therapies, there is a clear need for the development of new, alternative therapies with better efficacy and tolerability. New effective treatments would provide great benefit by reducing the health and economic burden of depression for patients, whānau (extended family), and the community at large.

### Microdosing of LSD

In lieu of more tolerable and efficacious treatments being available, we are now seeing a concerning trend worldwide for patients to forego conventional antidepressant therapies and instead self-medicate by “microdosing” psychedelic drugs such as LSD and psilocybin [8, 9]. Microdosing refers to the repeated consumption of LSD or psilocybin for weeks/months in doses below the threshold for causing pseudo-hallucinations [10]. In the last decade, the phenomenon of microdosing has emerged in an underground community of lifestyle drug users [11] with grey literature suggesting that this practice can improve mood [8, 10, 12].

Retrospective surveys of people who have microdosed consistently cite mental health improvements as both a principal motivation for, and outcome of, microdosing [9, 11–16]. One survey found that 39% of respondents were motivated by self-treatment of disorders including depression, anxiety, attention deficit hyperactivity disorder (ADHD), post-traumatic stress disorder (PTSD), and substance dependence [8]. Among these respondents, nearly 90% rated the practice as helpful and only 1.7% rated it as unhelpful, as opposed to antidepressants, which only 35.5% rated as helpful and 53.9% unhelpful. Studies tracking communities of people microdosing have shown, using validated subjective measures, significant increases in mental well-being and decreases in depression and anxiety over 4 weeks [13] and significant decreases in depression and stress symptoms over 7 weeks [15]. Although microdosers report self-medicating for various psychological conditions, depression appears to be the most common [15], and as such, depression is the most natural indication to test as a clinical application of LSD microdosing. While there has been extensive global public interest in microdosing as a potential therapy, research has not been able to keep pace with this interest. To our knowledge, the trial to be conducted here will be the first clinical trial of LSD microdosing in patients with MDD. The present study will attempt to determine whether a regimen of LSD microdosing is feasible and tolerable for individuals diagnosed with MDD.

### Safety of LSD microdosing

The trend for patients with psychological symptoms to self-medicate with psychedelic microdoses is both interesting and concerning. Although many studies indicate that LSD is relatively safe in terms of physical effects [17–21], the safety of microdosing in specific groups with mental health conditions is unknown.

Safety data from healthy volunteers is available from laboratory-based randomised controlled trials. In four papers with relatively minimal safety reporting, LSD microdoses were administered to approximately 138 participants in total [19–22] with no serious adverse events reported in these studies. Three of these studies have shown dose-dependent increases in blood pressure [19, 20, 22]. In a more thorough documentation of adverse effects, Family et al. [18] reported treatment-emergent adverse events (TEAEs) in 48 healthy older volunteers receiving six doses, one every 3 days, of either 0, 5, 10, or 20 μg of LSD. Although between 66.7 and 83.3% of participants in each group reported TEAEs, the only statistically significant difference between the groups was the frequency of headaches. The percentage of volunteers at LSD doses 5 μg, 10 μg, and 20 μg reporting headaches were 16.7%, 50.0%, and 25.0%, respectively, compared to 8.3% in the placebo group. All headaches were either mild or moderate. No change in vital signs was observed [18].

The phase 1 healthy volunteer (MDLSD) study we recently conducted using home administration of LSD microdoses similarly reported no serious or severe adverse events [17]. Adverse events included mild nausea, potential increases in jitteriness, stress, vivid dreams, and anxiety, but the increase in headache frequency reported by Family et al. [18] was not replicated in the MDLSD study—which had a much larger sample size. No changes in vital signs were detected.

### Scientific basis for current study design

The present microdosing study is based on the needs of the intended population of end-users and considers the common practices of microdosing in the community and results from the MDLSD trial [17]. It is an exploratory open-label phase 2a trial with the possibility for participants to take part in an extension period.

### Dosing protocol

The MDLSD dosing protocol of 10 μg, every third day was intended to closely mirror community microdosing practices, where doses are commonly self-reported as being between 10 and 13 μg [~ 10% of a full dose “trip”; 10, 23] and follow the highly popular Fadiman [10] schedule of dosing every third day. However, LSDDEP1 will consider some of the shortcomings of this scheduling and that of others to modify the dosing schedule to better suit the pragmatic study population’s lifestyle and scheduling considerations. Three key modifications have been made: (1) reduction of the initial dose, (2) dose titration, and (3) reduction of the number of doses a week.

The reduction in the initial dose to 8 µg along with dose titration has been informed by the results of the MDLSD trial [17]. Following the first wave of 19 participants in the MDLSD study, some participants reported feeling over-stimulated. A dose titration procedure was added to the protocol to reduce these unpleasant effects. Over the rest of the MDLSD study, seven participants entered the titration procedure (reduction to 5 μg then increase by 1 μg steps as feasible). Six were in the active intervention group and one in the placebo. Four participants completed the protocol under the titration protocol and three were withdrawn due to adverse events [17]. In the current study, the most appropriate dose for a participant will be the one in which they may feel subtle effects of the LSD, but not to the extent that is negative, overstimulating, or consciousness-altering. Thus, in LSDDEP1, the initial dose has been reduced to 8 μg, increasing at a rate of 1 µg per dose to a maximum dose of 15 µg. If participants experience any uncomfortable effects, their dose will be reduced by 3 µg for their next dose, with this increasing by 1 µg per dose to a level where they are comfortable. The decision to increase/decrease the dose will be based on the participant’s self-report.

Changes in dosing frequency made, come from a combination of both scheduling awkwardness and a lack of flexibility for participants. In practice, dose administration every third day misaligns with the 7-day week and is inconvenient for both participants and for study scheduling [17]. In this protocol, participants will microdose 2 out of 7 days a week for 8 weeks with the restriction that microdoses should not take place on consecutive days. As such, a 16-dose regimen will be used for the 8-week treatment period. Reduction in dosage frequency allows for flexibility for the participant and accommodation if they have a special event or need to undertake activities (e.g. driving) on certain days where the dosing may not allow them to.

### Participant safety

A number of safety aspects of the current trial have been carefully considered. A patient history of psychosis will be considered as contraindicated—although specific data on this is lacking. Participants with a family history of psychosis (confirmed first-degree relative) will be excluded from the trial. LSD, even in microdoses, has sympathomimetic effects and can cause increases in heart rate and blood pressure [20]; thus, participants with a significant history of cardiovascular disorders will also be excluded from this study. While there are no modern studies of potential teratogenic effects, LSD studies from the 1950s/1960s generally failed to find teratogenic effects of LSD, and no epidemiological studies have linked LSD to birth defects with the widespread community use of LSD [23]. Nevertheless, applying the precautionary principle, persons who are pregnant or lactating will be excluded from the current trial. Sexually active persons of child-bearing potential can be enrolled if they have a negative pregnancy test at screening and agree to use effective contraception for the duration of the clinical trial. Male participants must also agree to use effective contraception for the duration of the clinical trial. See Supplementary Participant Information Sheets for definitions of effective contraception. Special consideration is given to the impact of LSD on driving and the legality of the drug itself. As the effect of LSD microdosing on driving has not been investigated, participants will be instructed not to drive for the 6 hours following dosing. As LSD is a class A scheduled substance in New Zealand (Aotearoa), participants should only use the drug as instructed and will be made aware that misuse or supply to others constitutes a criminal offence that may be prosecutable.

### Justification for concurrent antidepressant use

In a perfect world, all potential participants would be free of antidepressant medications providing a “clean” sample of participants to enrol study in phase 2 studies. In reality, this is not the case as many adults in Aotearoa with MDD have been prescribed an antidepressant of some kind [24]. Even if we were able to sample currently unmedicated patients as we did in another study [25], this sub-population would probably not be representative of the wider MDD population.

Commonly, participants entering a clinical trial will have a medication washout prior to trial enrolment (often defined as being 2 weeks medication-free). However, these protocols are likely inadequate with NICE UK [26] recommending antidepressants are tapered off over a 4-week period. NICE also note that discontinuation symptoms can occur up to 9 weeks after cessation, with the potential for participants to be de-stabilised, with worsening depression [27], representing a safety issue. Moreover, patients with less stable depression are more likely to show regression to the mean effects and overall higher variance in the main efficacy measures, particularly the MADRS which would be counter-productive to the purposes of the LSDDEP1 study.

Most antidepressants are unlikely to present a safety issue to participants when taken alongside LSD microdoses; however, antidepressants might dampen the response to LSD [28]. Given that the current trial will use dose titration, in theory at least, this might to some extent ameliorate this dampening issue. As such, this trial will allow participants to maintain a stable antidepressant therapy while in the trial (only excluding monoamine oxidase inhibitors).

### Cultural considerations

It is essential that any work undertaken in Aotearoa fully considers the impact on and for the indigenous Māori population. This is especially the case for mental health research, as Māori tend to be overrepresented in prevalence statistics [17% New Zealand European vs. 21% of Māori diagnosed with MDD between 2020 and 2021 [29] and experience worse outcomes with the healthcare system [30]. This gives a strong long-term goal of this research and many others to provide equal access to healthcare and improved mental health outcomes for Māori individuals.

The disproportionate number of Māori individuals suffering in the mental health sector fortifies the idea that Māori should be involved in each step of the research process to provide the best possible outcome. Māori consultation has been undertaken with the Aotearoa Psychedelic Māori Advisory Rōpū (group) (AP-MAR) regarding the implementation of LSDDEP1 and will be ongoing throughout the research process. The AP-MAR group includes Māori advisors, researchers and psychologists who carefully assess proposals to ensure Māori benefit from the development of interventions and that they are culturally safe and sensitive. Te Ao Māori (Māori worldview) aspects have been woven into the design and assessments including the use of Hua Oranga to assess the domains of a widely used Māori model of health, Te Whare Tapa Whā [31], and various aspects of the protocol will be implemented in such a way as to ensure that Tikanga (customary practices) are observed.

One major shortcoming of the MDLSD trial conducted was that only 3.75% of the sample identified as Māori, while they represent 16% of the population of Aotearoa. To remedy this under-sampling, the current protocol will ensure that a minimum of 25% of participants identify as Māori. Another aspect concerning Māori is the importance of encouraging participants to bring in whānau (extended family) to support them in assessments and dosing days. We will also be undertaking whānau interviews after the completion of the trial to see what they thought about the trial and any changes they noticed in the participant (their whānau).

### Patient public involvement panel

The current clinical trial design was produced in consultation with a patient and public involvement panel of persons with lived experience with depression using the UK INVOLVE standards [32]. This process began with a public forum on the LSDDEP trials attended by approximately 100 people in person and via video conference with 12 patients later invited to join the panel. At the date of submission, three panel forum meetings have been held. In particular, panellists clearly endorsed/encouraged (a) the overall study design, (b) the concurrent use of antidepressants, and (c) the use of extension periods for fairness. Panellist involvement is anticipated to continue throughout the life cycle of LSDDEP1.

### Objectives

The primary objectives of LSDDEP1 are to assess the tolerability of the designed regimen of LSD microdoses in patients with MDD and to assess the feasibility of conducting a larger RCT using the described study procedures. Tolerability will be assessed by quantifying the percentage of participants who withdraw from the trial due to adverse events attributable to the treatment regimen, while feasibility will be measured as the percentage of attended clinic visits once a patient is enrolled in the trial.

Secondary objectives will also be examined within this study to give some clinically relevant information. Specifically, this will be to track the time course of depressive symptomology in patients with MDD receiving the proposed regimen of LSD microdoses using the Montgomery-Asberg Depression Rating Scale [MADRS; 34]. LSDDEP1 is being conducted in anticipation of the conduct of LSDDEP2, a subsequent randomised control trial with the same or similar dosing regimen and a larger participant group. A further secondary goal is to measure compliance with the trial assessment load given the large number of exploratory measurements that will be conducted (described in the “ Outcomes” section). Overall, the objective of LSDDEP1 is to pilot and optimise trial procedures for LSDDEP2.

## Methods/design

### Participants

There will be 20 participants diagnosed with MDD as per the Diagnostic and Statistical Manual of Mental Disorders, Fifth Edition (DSM-5) criteria for MDD as identified by clinical interview. At least 25% of the sample will be Māori. Participants will be required to meet all the inclusion and exclusion criteria outlined in Tables 1 and 2 and adhere to the lifestyle considerations outlined in Table 3.

**Table 1:** Full inclusion criteria

	Inclusion criteria
Consent	Provision of signed and dated informed consent form Stated willingness to comply with all study procedures and availability for the duration of the study For sexually active persons of child-bearing potential, i.e. assigned female at birth: agree to use an effective or highly effective contraception for at least 1 month prior to screening and agreement to use such a method during trial, until the one-month follow-up is completed For those assigned male at birth who are of reproductive potential: use of condoms or other methods to ensure effective contraception with partner Ability to take oral medication and be willing to adhere to the study intervention regimen
Demographics	Any gender identity Aged, 21–65 years
Clinical characteristics	Diagnosis of MDD as per the DSM-5 criteria for MDD (determined by clinical interview) • Have a MADRS score between 18 and 35 at the time of screening

**Table 2:** Full exclusion criteria

	Exclusion criteria
Mental health diagnosis	Current or past history of schizophrenia or other psychotic disorders or bipolar I or II disorder as assessed by clinical interview. Patients with MDD with psychotic features will be excluded. Also excluded will be individuals with a known first-degree relative with these disorders. Diagnosis of PTSD Diagnosis of an eating disorder
Current risk	Stage II or higher treatment-resistant depression as defined by the Thase and Rush [33] staging criteria for the current depressive episode. Risk of suicide as determined by The Columbia-Suicide Severity Rating Scale (C-SSRS). Specifically, patients answering “yes” to items 3–5 covering the last 3-month period will be excluded.
Drug use	Substance dependence in the previous 6 months use as assessed by clinical interview with a New Zealand modified version of the NM-ASSIST. Problematic use of alcohol defined as a score on the AUDIT of 16 or greater. Use of monoamine oxidase inhibitors, methylphenidate, or dexamphetamine. Excessive ongoing medication burden as determined by a study physician. Regular use of any medications/supplements deemed to be contraindicating as judged by a study physician. Treatment with another investigational drug or other intervention within 2 months. Any lifetime history of psychedelic microdosing. Use of serotonergic psychedelic drugs (LSD, psilocybin, DMT, etc.) in the last year. Lifetime history of self-medicating with psychedelics to treat their depression.
Physical health	BMI < 18 and > 35. Planned or current pregnancy or lactation.
Vital signs	Cardiovascular conditions including abnormal heart rate or blood pressure to be checked at screening. A threshold of exceeding 160 mmHg (systolic) and 90 mmHg (diastolic), averaged across three assessments taken on the screening day will be used. Participants with well-managed hypertension will not be excluded.
Laboratory tests	Significant renal or hepatic impairment. Abnormal 12-lead ECG as judged by a study physician. Abnormal laboratory test findings (complete blood count, liver function test, renal function test, thyroid function test) as judged by a study physician.
Diagnoses	Any unstable medical or neurological condition. Any other condition judged by the treating clinician as likely to impact on the ability of the participant to complete the trial.

**Table 3:** Lifestyle considerations

	Lifestyle considerations
Caffeine	Limit caffeine consumption to ~ 100 mg on dosing day.
Alcohol	Abstain from alcohol for 24 h before the start of each first dosing session. A breathalyser test will be performed at each dosing session. A failed breath alcohol test (> 0 µg/L breath) will lead to withdrawal.
Recreational drugs	Abstain from recreational drugs for the duration of the study.
Tobacco	Participants who use tobacco products will be instructed that use of nicotine-containing products (including nicotine patches) will not be permitted while they are at the study site.
Depression therapy	Not begin any new therapies for depression over the course of the study.
Menstruation	For persons who are menstruating, best efforts will be made to time the dosing session with the start of the follicular phase of the menstrual cycle, and participants will be asked to report the onset of menses during their participation.

### Study design

The study is a phase 2a open-label pilot trial of LSD microdosing in patients with major depressive disorder (MDD) to test the feasibility and tolerability of trial procedures for a subsequent randomised controlled trial (LSDDEP2). Eligible participants (*N* = 20; ≥ 5 Māori) will all receive LSD microdoses, beginning at 8 μg with a titration range from 5 to 15 μg. The LSD will be self-administered (for more information see the “ Drug preparation and administration” section). All “on-site” visits will occur at the Clinical Research Centre on the Auckland University Grafton Campus in Auckland, New Zealand.

Upon expression of interest in the trial, participants will be emailed a link to a short pre-screening questionnaire. This allows for rapid determination if participants would be excluded and reduces the burden on an already distressed population if they are not able to progress to the next stage. Following completion of the pre-screening questionnaire, eligible participants will be emailed the relevant participant information sheet (PIS) and consent form (CF) (see Additional file 1) and invited to take part in the screening process. The PIS will be given to potential participants before their screening visit, allowing them enough time to seek independent counsel, such as that from a lawyer, general practitioner (GP), or family member. These documents contain information on the trial’s purpose, what will be asked of the participant, known risks of participation, and any implications and constraints of the protocol. Participants will be allowed and encouraged to ask questions of the study team at any point prior to or during the screening process.

The screening process will be split into two sessions, the first of which will occur remotely over phone/video call for participant convenience. This will involve obtaining medical and psychiatric history, carrying out the MADRS and C-SSRS and assessment of inclusion/exclusion criteria (see Tables 1 and 2). Both the MADRS and C-SSRS have been endorsed for remote use [34]. At this stage, the research team will ensure that the participant knows the time requirements, risks, and assessments required for the trial and informed consent will be confirmed via their verbal understanding of the information presented and through written, e-signed informed consent. Verbal consent and eligibility will be confirmed at each session. If eligible, participants will be invited to an in-person screening. This will occur onsite and include physical measurement of height, weight, vital signs, 12-lead ECG, laboratory tests, drug/alcohol breathalyser test, and pregnancy test.

**Fig. 1:**
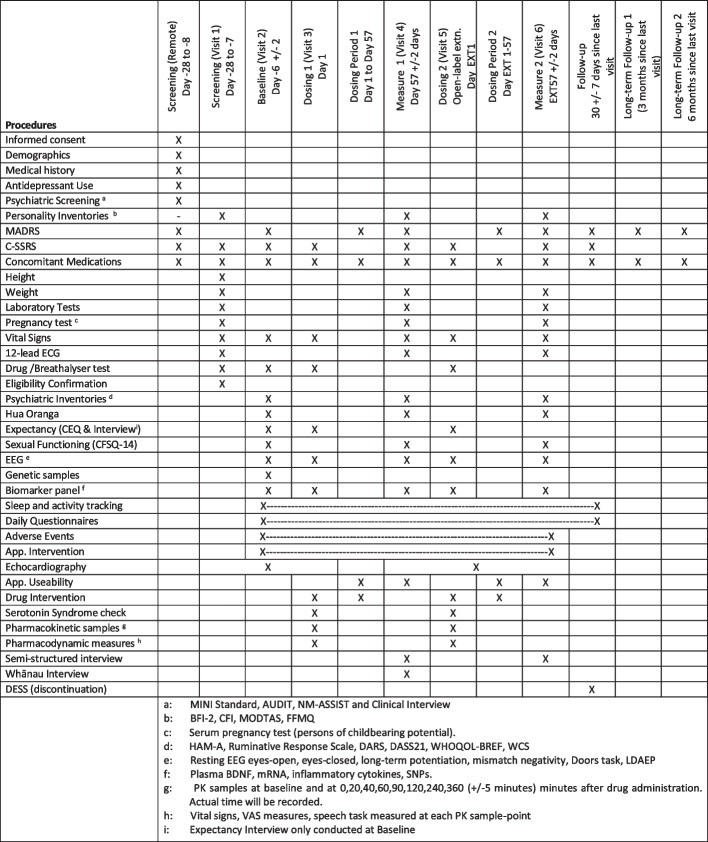
Schedule of activities

If participants are eligible after the screening battery, they will be invited to attend a baseline session onsite (baseline visit; see Fig. 1). This will involve baseline electroencephalography (EEG) readings, MADRS and other depression inventories, and providing blood samples for the biomarker panel. At this stage, participants will be given a wearable activity tracking and a study mobile phone app will be installed on their phone. If a participant does not have a mobile phone, one will be provided for them. At this stage, adverse event (AE) recording, sleep and activity tracking, and daily questionnaires will commence. Echocardiograms will also be measured at a subcontracted clinical facility.

Six days (± 2) later, participants will be asked to come onsite again for their first dosing day (dosing 1; see Fig. 1). Participants will be given their first single dose of the investigational medicinal product (IMP) and be monitored for 6 h before being discharged. Blood will be drawn prior to drug administration and at 20, 40, 60, 90 120, 180, 240, and 360 min (± 5 min) after administration. Subjective drug effect measures (VAS scales) will also be collected at these time points as per Murphy et al. [17]. EEG measures will be commenced ~ 2 h after administration. Participants will then be discharged with five doses for additional dosing. These doses will be self-administered sublingually two out of every 7 days. Participants will attend a further two re-supply visits during the dosing period. Between days 1 and 57 (dosing period 1; see Fig. 1), they will undertake at-home dosing 2 times a week for a total of 16 doses over 8 weeks.

During this period, they will complete a MADRS on days 14, 28, and 42 (± 3 days) over a video call. Participants will then be asked to come in for their fourth and final visit on day 57 (± 2 days). In this visit, they will undertake a final MADRS, EEG, biomarker panel, and semi-structured interview. If they consent to, at this stage, a whānau (extended family) member will also be asked to attend a separate semi-structured interview considering their whānau member’s experience with the trial (for the full schedule of activities, see Fig. 1). At the discretion of investigators, participants in this trial will have the option to undertake an up to 8-week extension period with the same protocol as the initial part of the trial. Participants will also have three follow-ups, one at 30 days (± 7 days) from the last measure session date and one at 3 months and 6 months from the last measure date.

### Outcomes

The primary and secondary outcome measures of this trial are displayed in Tables 4 and 5, respectively. The primary measures are tolerability and feasibility. Tolerability is measured through the percentage of participants who withdraw from the trial due to adverse events attributable to the treatment regimen where a lower value represents better tolerability. Feasibility will be measured via compliance with the LSDDEP1 protocol, detailed by the percentage of attended clinic visits once enrolled. Both of these measures, alongside participant feedback throughout the trial and in the semi-structured interviews, will allow for the optimisation of procedures for the subsequent LSDDEP2 trial. The full schedule of assessments is detailed in Fig. 1.

**Table 4:** Primary measures

Outcome domain	Measure	Definition
Tolerability	Adherence to a regimen of LSD microdoses	Percentage of participants who withdraw from the trial due to adverse events related to the treatment regimen
Feasibility	Compliance with LSDDEP1 study procedures to determine the feasibility of conducting LSDDEP2	Percentage of attended clinic visits once enrolled

**Table 5:** Secondary measures

Outcome domain	Measure	Scale
MDD symptoms	MADRS [35] assessed at baseline and at 2, 4, 6, and 8 weeks time points	10 items, each clinician-rated on a 7-point Likert scale, summed to give a total score between 0 and 60
Compliance	Acceptability of trial assessment load	Percentage of assessments completed by assessment type

The participants will also undertake a battery of psychological testing which includes the MADRS which is a secondary outcome in the present study, however, the MADRS will be used as the main efficacy measure for LSDDEP2. The MADRS is one of the most commonly used depression scales in pharmaceutical/regulatory registration trials of depression and consists of 10 items which are summed to a maximum potential score of 60 [36]. Compliance with the trial load will also be quantified as the percentage of assessments completed for each assessment type and time point. This will provide a measure of the acceptability of the trial assessment load, which is important as we do not want to overburden the depressed patient sample. Both the MADRS and compliance will be secondary measures of this trial seen in Table 5.

Safety measures are detailed in Table 6 and include recording of adverse events, measurement of objective safety measures, and checks for serotonin syndrome. Adverse events and their severity will be recorded by the participant in the study app off-site and by the research team on-site. Measures of objective safety will include complete blood count, liver function, renal function, thyroid function, 12-lead ECG, and vital signs at baseline, at the 8-week time point and after completion of the extension period. Echocardiograms will be recorded at baseline and after maximum drug exposure depending on whether a participant enters the extension period. Serotonin syndrome checks will be made on dosing 1 and dosing 2 visits, consisting of checks of body temperature, diaphoresis, shiver/tremor, and agitation and induced clonus if indicated.

**Table 6:** Safety measures

Outcome domain	Measure	Scale
Adverse event profile	Assess the incidence of SAEs and AEs by severity, recording in app and on site	Tabulations of AEs by severity and SAE listings from baseline to 1 month post-intervention.
Modification of objective safety measures	Laboratory tests	Complete blood count, liver function, renal function, thyroid function), 12-lead ECG, and vital signs will be measured at baseline and at the 8-week time point and after completion of the extension period. Echocardiogram will be measured at baseline and after the last dose is taken.
Serotonin syndrome	Complete serotonin syndrome checks at each dosing visit	Clonus, diaphoresis, shiver/tremor, and agitation body temperature.

A number of additional exploratory measurements will also be made; for the full description, see Table 7.

**Table 7:** Exploratory measures

Outcome domain	Measure	Scale
Anxiety symptoms	HAM-A [37] assessed at baseline and at the 8-week time point.	14 items, clinician-rated on a 4-point Likert scale; these scores combined to give a final composite score.
Depression, stress, and anxiety symptoms	DASS-21 [38], assessed at baseline and at the 8-week time point.	21 items, 5-point Likert scale, from 0 (never) to 4 (almost always). Three subscales reported as summed scores.
Anhedonia symptoms	DARS [39], assessed at baseline and 8-week time point.	4 domains that call for participant examples, rated on a 5-point Likert scale (0–4). Total and each of its four subscales will be compared.
	EEG doors paradigms [40] will be measured at baseline, dosing, and measure time points.	Trials where participants are presented with two doors to select from. In half the trials, they receive feedback indicating monetary gain or loss. Positive ERP ~ 250–300 ms after feedback.
Ruminative symptoms	RRS [41], assessed at baseline and at the 8-week time point	22 items, four scales measuring two aspects of rumination, rated from 1 (almost never) to 4 (almost always).
Quality of life	WHOQOL-BREF [42], assessed at baseline and 8-week time point	26 items, scored on a 5-point Likert scale. Each of its four subscales will be compared.
Connectedness	Watts Connectedness Scale [43], assessed at baseline and 8-week time point.	19 items marked on a visual analogue scale between 0 (“not at all”) and 100 (“entirely”). Each of the three subscales will be compared.
Te Whare Tapa Whā	Hua Oranga [31, 44], assessed at baseline and 8-week time point. Each of its four subscales will be compared.	16 items where each of the constructs is scored by participants on a 5-point scale with descriptors of each provided. Each of its four subscales will be compared.
Daily mood	HAMD-6 self-report [45] will be assessed from baseline daily through until follow-up 1.	6-item scale, clinician-rated. Captures core features of depression.
EEG	The EEG long-term potentiation (LTP) [46, 47], mismatch negativity (MMN) [48], loudness-dependent auditory evoked potential (LDAEP) [49], as well as resting state will be measured at baseline, dosing, and measure time points.	Recorded with 64 channel caps, 5 min eyes-open 5 min eyes-closed resting state, sensory LTP with vertical and horizontal sine gratings on grey background, MMN 5–11 sinusoidal tones, classic oddball response, LDAEP measures serotonergic function, 100-Hz stimulus tones at differing intensities.
Plasma/serum BDNF and mRNA biomarkers	Plasma/serum BDNF samples will be measured baseline. Dosing (pre and 6 h post) and measure time points. Buffy coats will be taken for mRNA samples.	BDNF plasma levels, expression of mRNA markers including proBDNF, BDNF, TrkB, p75NTR, sortilin, 5HT2A, and 5HT1A.
Genotype prediction of outcome measures	Whole blood samples will be taken from participants at baseline.	ValMet66 polymorphism of BDNF gene, SNP analysis related to CYPs and 5HT2A receptor.
Personality trait modification	BFI-2 [50], CFI [51], FFM-Q [52], and MODTAS [53] are measured at screening and measure points.	BFI 60 items on Likert 1–5, for the main personality traits, CFI 20 items from 1–7 giving total score and 2 subscales, FFM-Q 38 items on a 5-point Likert scale, and 34 items on a 5-point Likert scale.
Expectancy	CEQ [54] and short expectancy interview will be measured at baseline and dosing sessions.	6-item scores on a 9-point Likert scale.
Sexual dysfunction	CSFQ-14 [55] will be measured at baseline and measure sessions.	14 items, rated on a 5-point Likert scale.
Sleep, activity, and physiology	Fitness tracker data will be recorded continuously from baseline through to 1 month after completion of regimen.	Wearing of a Garmin activity tracker logging basic measurements including sleep duration, quality, physical activity, heart rate, and stress levels
Daily experience	Daily VAS scales completed every evening in the study app. Participants will be encouraged to record an audio journal in the study app.	HAMD-6 self-report with VAS scales for connected, creativ﻿e, energy, happy, irritable, and jittery. Adverse event reporting.
Withdrawal/discontinuation symptoms	DESS [56] will be measured at the follow-up 1 time point.	Ch﻿eck﻿s for symptoms of discontinuation syndrom﻿e.
Acceptability of study app	Acceptability questionnaire completed every 2 weeks.	5 items considering usability of the app.
Engagement with therapeutic journaling	Journal entries.	Number of journal entries submitted accessed via the app.
Subjective experience	A semi-structured interview.	Conducted with the participant (and whānau member) to record their subjective experiences of being involved in the trial.
Plasma pharmacokinetics	Pharmacokinetic samples will be taken at baseline and 20, 40, 60, 90, 120, 180, 240, and 360 min (± 5 min) after the first dose (dosing).	Quantification by liquid chromatography mass spectrometry
Pharmacodynamics	Pharmacodynamic measurements will be recorded at baseline and at 20, 40, 60, 90, 120, 180, 240, and 360 min (± 5 min) after the first dose (dosing).	Vital signs, speech task, and VAS scales.
Extension period	An intervention extension period is offered to all patients for 8 weeks.	The number of doses administered in this period is the endpoint.
Durability of antidepressant response	MADRS [35] assessments will be conducted at three follow-up time points (1, 3, and 6 months after the final measure session).	10 items, each clinician-rated on a 7-point Likert scale, summed to give a total score between 0 and 60.

### Participant recruitment

This trial will aim to recruit 20 patients, at least 25% of who will identify as Māori. Participants may need to be excluded based on ethnicity basis if recruitment targets have not been met.

All participants will be recruited from the New Zealand community. Participants will be recruited from general practices within the greater Auckland area and via advertisements placed in local newspapers, noticeboards, and online using social media, allowing potential participants to make initial contact with the study team. Our study team also has a database of patients with (self-reported) major depressive disorder who have expressed interest in participating in clinical trials. These patients will be directly emailed by the study team. Participants will first complete a brief pre-screening questionnaire for trial staff to allow rapid determination if the participant would be excluded.

If eligible, participants will receive koha (gift) in recognition of the time and commitment associated with taking part in the study. Individuals will receive a total of $250 in gift cards at the end of the study and have any expenses reimbursed. In addition, participants will receive a further $20 each time they complete the doors EEG task. Participants who fail screening will be given a $20 gift card for their time.

### Strategies to improve adherence

The home-dosing protocol will be monitored through mobile directly observed therapy (MDOT) to ensure adherence and prevention of medication stacking. MDOT has been employed in various medication scenarios such as the utilisation of asthma inhalers [57], and the MDLSD trial confirmed 100% compliance with a regimen for that trial [17]. On dosing days, participants will receive an app notification reminding them to take their medication that morning and asked to record a video of them taking the medication.

Using their mobile phones, participants will record a video clearly showing the self-administration procedure. The trial staff will check the video for compliance with instructions. If a participant repeatedly performs the MDOT procedure poorly, they will be removed from the trial at the discretion of an investigator. Videos will be deleted immediately after compliance checking and noted in the electronic case report form (eCRF). Participants will be trained on the MDOT procedure during the large break periods of dosing 1.

### Drug preparation and administration

Good Manufacturing Practice (GMP) quality LSD Hemitartrate Active Pharmaceutical Ingredient (Psygen Ltd., Calgary, Canada) will be formulated to GMP by Biocell Corp. (Auckland, New Zealand) to produce MB-22001—the investigational medicinal product (IMP) to be used in this trial. Doses are stated as free base equivalents in this document. The contract manufacturer will receive a MedSafe Manufacturing licence for the IMP prior to the commencement of the trial. Investigational products will be labelled consistent with legal requirements. All participants will be offered a lock box to securely keep the IMP at home—and to prevent accidental ingestion by minors or individuals other than the participant. MB-22001 is a liquid formulation that participants can self-administer sublingually.

Participants will be supplied up to a maximum of five LSD doses at a time. The dosing regimen includes 16 doses with the first dose given in clinic on dosing day and subsequent doses self-administered twice a week for 8 weeks The first box of five doses will be supplied at this visit. A further two re-supplies will need to take place over the dosing period. Participants will be asked to dispose of packaging and any residual dose themselves.

Instructions for self-administration will be guided by the study mobile phone application. Doses should be taken no later than 2 pm each day to prevent disruption to sleep. Participants are instructed not to drive or engage in any dangerous activities for a 6 hour window following dosing.

### Psychotherapeutic element

In the grey literature, self-medicating microdosers recommend setting intentions for activity and reflecting on the acute microdosing experience to improve the purported benefits. MDLSD qualitative reports appear consistent with this (data in preparation). As such, participants will be encouraged to take part in activities such as walking, engaging with creative pursuits, and social activities, and the drug intervention will be accompanied by a psychotherapeutic intervention to try to maximise the potential psychological effects of microdosing. Delivery of this intervention will be standardised and programmed into the study mobile phone app. For each microdosing session (apart from the first dose in the laboratory), participants will be asked to choose an activity to do on the microdosing day. At the baseline session, an initial set of activities will be selected with assistance from a member of the trial team and loaded into the app on the participant’s mobile device. During the dosing session, the app will remind the participant of their scheduled activity. On the evening of the microdose, participants will then be able to journal and reflect on their day with prompts provided by the app. Journaling can be done via, audio, text, or photo (of handwritten entries) at the participant’s discretion. The number of journal entries submitted will be an outcome measure.

### Extension period

LSDDEP1 will have an optional extension period lasting up to 8 weeks. This will allow preliminary investigation of the desire and effects of participants to continue the intervention regimen. In the extension, participants will be allowed to dose less than twice a week should they choose to and this would not be considered a protocol violation. Commencement of the extension period can be delayed by up to 14 days from measure 1 visit. Should a participant wish to stop microdosing halfway through the extension period they will be invited in for a final measure session and then enter the follow-up period.

### Relevant concomitant care and post-trial care

Throughout the research, participants will get care as usual from their general practitioner and will be provided recommendations for preferred therapies for any non-exclusionary health conditions that arise. It is considered exceedingly unlikely that participants will suffer long-term harm; however, participants will be able to apply for compensation for any injury sustained during the trial under the University of Auckland insurance policy.

### Statistical analyses and power calculations

No frequentist inferential statistics will be performed on data collected from this pilot study to inform the transition to LSDDEP2. Analyses will consist of descriptive statistics such as means, medians, standard errors, and 95% confidence intervals. The main descriptive statistics will be the percentage of participants completing the dosing regimen; percentage of attended clinic visits once enrolled; percentage of measures completed grouped by measure; change in MADRS scores 2, 4, 6, and 8 weeks of LSD microdosing compared to baseline; and percentage of participants classified as responders and remitters at 2, 4, 6, and 8 weeks as measured by the MADRS. A “responder” at a particular time point is defined as a participant who experiences a 50% reduction in MADRS score relative to baseline. A “remitter” at a particular time point will be classified as a participant who has a MADRS score < 10 at that time point [58].

This is the first study to investigate LSD microdosing as an intervention for MDD, and as such, there are no previous effect size estimates on which to base power calculations. Given the desire to also explore secondary and exploratory outcomes with maximum power, the sample size was based on pragmatic reasons (cost and potential ability to recruit participants).

### Adverse event reporting and harms

An adverse event (AE) is any unexpected medical occurrence (e.g. any unfavourable and unintended sign, including abnormal laboratory or physical exam findings, symptoms, or disease) that arises during participant involvement in the research whether or not they are considered to be related to participation. This definition includes concurrent illnesses, injuries, and exacerbation of pre-existing conditions. It does not include anticipated day-to-day fluctuations of pre-existing disease(s) or condition(s) present or detected at the start of the study that do not worsen. As such, any AE may be temporally or causally associated with the use of the investigational medicinal product.

Adverse events occurring off-site will be recorded via the mobile phone app. Participants will be prompted to describe the event, give a date and time of onset, request a follow-up call from the study team if desired, and give a self-graded severity of the event (mild, moderate, or severe). AEs recorded in the daily report form will be reviewed daily by trial staff. Any AE rated as moderate/severe by a participant will result in an immediate alert to the study team.

Adverse events occurring onsite will be recorded in the case report form (CRF), whether or not they are attributed to trial medication. AEs will be actively sought through non-directive questioning of participants at each study visit. Additionally, patients may voluntarily report adverse events during or between visits, and they can also be identified through physical examination, laboratory tests, or other assessments. AE information recorded in the CRF includes description, date and time of onset, severity grade (mild, moderate, or severe), and the relationship between AE and IMP (related or not related), if the AE is expected or unexpected, action taken and outcome.

All serious adverse events (SAEs) will be reported to MedSafe by the principal investigator as per section 6 of the MedSafe reporting guidelines described in the Regulation of Therapeutic Products in New Zealand Part 11: Clinical trials – regulatory approval and good clinical practice requirements. These are submitted via the HDEC website (https://nz.forms.ethicalreviewmanager.com) as soon as is practical.

### Participant withdrawals

If a participant requests it, one of the exclusion criteria listed in Table 2 above is violated, there is insufficient dose compliance (at the discretion of study investigators), they experience a serious adverse event or if any other condition arises that the study team determines is likely to have an impact on their ability to function, and the intervention will be stopped immediately. Decisions to withdraw a participant will be made at the discretion of the study clinicians.

### Data collection and management

Data collection is the responsibility of the clinical trial staff at the site under the supervision of the study investigators. Individual paper-based files will be maintained for each participant, while the majority of the CRF and data capture will be handled through the online Research Electronic Data Capture (REDCap) tools hosted at the University of Auckland. REDCap is a secure, web-based software platform specifically designed to facilitate data capture for clinical trials.

Clinical data (including AEs, concomitant medications, expected adverse reactions data), demographics, medical history, height, weight, notes on physical examinations, alcohol/drug screening results, vital signs, eligibility confirmation, self-reported questionnaires, and daily questionnaires will be entered into REDCap. All electronic data is to be stored on password-protected University of Auckland servers that have multi-site backups and tape archiving. Each data file will have a corresponding original, unprocessed version that can only be modified by a University of Auckland IT systems administrator, thus ensuring audit capability and accuracy of extracted data.

For all data, participants will have a unique trial number which will be recorded on all their electronic forms. On all trial-specific documents excluding the signed consent, prescriptions, referral forms, labelled IMP, and page one of the CRF, participants will be referred to by this number, not their name. All source data and study documents will be held for a period of 15 years from the completion of the trial.

### Trial governance

The Trial Steering Committee (TSC) consists of a subset of investigators from the study who will oversee the trial. In particular, the TSC will collaboratively develop and approve the final protocol; oversee the progress of the trial, adherence to the protocol, participant safety and consideration of new information, and be responsible for publication and dissemination. The TSC was in full agreement prior to submission of the final protocol and will take responsibility for major decisions, e.g. change of protocol, supervision of trial progress, and reviewing relevant information from other sources. If a change is necessary, a minimum agreement of 50% of the TSC, including the PI, is required, with the PI holding the deciding vote. No external independent Data Monitoring Committee will be formed for this pilot trial. Clinical site monitoring will be conducted by the National Institute of Health Innovation (www.nihi.auckland.ac.nz).

### Dissemination policy

This study will be registered at ANZCTR, and result information from this trial will be available within 12 months of completion of the study. Results will be published in relevant academic journals and communicated with the wider public via news media and social media.

## Discussion

The present study gives one of the first investigations into the potential effects of self-administered psychedelic microdosing in a depressed participant population over a long period in a naturalistic setting with LSD. The protocol entails an exhaustive battery of subjective psychological measures, objective laboratory testing, and qualitative interviews. It is not expected that these data by themselves will answer particular hypotheses at this stage; however, they do represent a prospective set of procedures and outcome measures that could be employed in LSDDEP2—a larger randomised controlled trial. One aim of the current study is to ensure that the many measurements taken do not place an excessive burden on participants as this could lead to poor data quality or a high level of dropouts—which would negatively affect an RCT. Similarly, the current intervention design of both pharmacological and non-pharmacological aspects has been based on community practises and findings from the phase 1 MDLSD study. Nevertheless, it is noteworthy that the demography of that dataset was healthy male volunteers, while the current participant population includes all gender identities with major depressive disorder. Objective data and qualitative interviews with participants may help to determine whether this intervention is appropriate for the study population and/or if further optimisations are required.

Together the LSDDEP trials will help to establish the effects of microdosing in a population sample of individuals experiencing depression. It is noteworthy that several uncontrolled studies of microdosers have suggested that many, if not all, of the claimed effects of microdosing are placebo responses [13, 59]. If this is the case, and microdosing can be shown to be purely placebo in terms of antidepressant effects, then it is important that this is established in the context of clinical trials. Patients would then likely benefit from returning to evidence-based usual care pathways, instead of self-medicating. On the other hand, if the antidepressant effects of LSD microdosing are confirmed, then appropriately regulated LSD microdosing regimens could be further developed as treatments.

## Trial status

The LSDDEP1 trial protocol is currently on version 2.3 (19 July 2023). Recruitment for this trial has commenced on 19 July 2023.

### Supplementary Information

**Additional file 1. **Participant information sheet and informed consent form.

## Data Availability

The corresponding author will release documentation including PIS, consent forms, and study advertisements on publication of trial results. Access to the final trial dataset will only be available to the study investigators and any other relevant regulatory bodies.

## Abbreviations

ADHD: Attention deficit hyperactivity disorder
AE: Adverse event
AUDIT: Alcohol Use Disorders Identification Test
BDNF: Brain-derived neurotrophic factor
BFI-2: Big Five Inventory-2
BMI: Body mass index
CEQ: Credibility and Expectancy Questionnaire
CF: Consent form
CFI: Cognitive Flexibility Inventory
CFSQ-14: Changes in Sexual Functioning Questionnaire short-form
CRF: Case report form
CSSR-S: Columbia-Suicide Severity Rating Scale
DASS-21: Depression, anxiety
DESS: Discontinuation emergent signs and symptoms
DMC: Data Monitoring Committee
DSM-5: Diagnostic and Statistical Manual 5
ECG: Electrocardiogram
eCRF: Electronic case report forms
FDA: Food and Drug Administration
GMP: Good Manufacturing Practice
GP: General practitioner
HAM-A: Hamilton Anxiety Rating Scale
HDEC: Health and Disability Ethics Committee
HRC: Health Research Council
IMP: Investigational medicinal product
LSD: Lysergic acid diethylamide
LTP: Long-term potentiation
MADRS: Montgomery-Ãsberg Depression Rating Scale
MAOI: Monoamine oxide Inhibitor
MDD: Major depressive disorder
MMN: Mismatch negativity
MDOT: Mobile directly observed therapy
NICE: National Institute for Care and Excellence
NM-ASSIST: NIDA (National Institute of Drug Abuse) Modified Alcohol, Smoking and Substance. Involvement Screening Test
OL: Open-label
PD: Pharmacodynamic
OCD: Obsessive Compulsive disorder
PI: Principal investigator
PIS: Participant information sheet
PK: Pharmokinetic
PTSD: Post-traumatic stress disorder
QOL: Quality of life
REDCap: Research Electronic Data Capture
RRS: Ruminative Response Scale
SAE: Serious adverse event
SCOTT: Standing Committee on Therapeutic Trials
SNRI: Serotonin and norepinephrine reuptake inhibitor
SOP: Standard operating procedure
SSRI: Selective serotonin reuptake inhibitor
TMF: Trial Master File
TSC: Trial Steering Committee
VAS: Visual analogue scales
WCS: Watts Connectedness Scale
